# Evaluation of a caudal midline glossectomy on tongue volume and upper airway cross-sectional areas in brachycephalic dogs: a cadaveric study

**DOI:** 10.3389/fvets.2025.1607711

**Published:** 2025-08-04

**Authors:** Valeria T. Colberg, Raymond K. Kudej, Nicole Moyer, Joshua A. Peters, William M. Karlin

**Affiliations:** Department of Veterinary Clinical Sciences, Tufts University, Cummings School of Veterinary Medicine, North Grafton, MA, United States

**Keywords:** BOAS, brachycephalic, brachycephalic airway obstruction syndrome, macroglossia, glossectomy

## Abstract

**Introduction:**

Relative macroglossia may contribute to brachycephalic obstructive airway syndrome, the pathologic disorder associated with respiratory dysfunction commonly seen in brachycephalic dogs. Recent studies on brachycephalic dogs have demonstrated a relative macroglossia along with reduced air volume in the upper airway compared to non-brachycephalic dogs. Tongue reduction glossectomy may be a surgical option to address upper airway obstruction secondary to macroglossia. The objective of this study was to evaluate the effects of a caudal midline glossectomy (CMG) on tongue volume and upper airway cross-sectional areas.

**Methods:**

Cadaveric brachycephalic dogs (*n* = 6) were positioned with the tongue retracted and jaw nearly closed. Computed tomography was performed to evaluate tongue volume and cross-sectional areas of tongue, oropharynx, palatal soft tissue and nasopharynx at two levels, the caudal aspect of the hard palate and pterygoid hamulae. A standardized CMG was performed. Positioning and CT scan were repeated.

**Results:**

CMG resulted in a 20% decrease in tongue volume (from 87,546 ± 21,121 to 70,259 ± 17,586 mm^3^; *p* < 0.01). CMG resulted in a 20 to 25% decrease in cross-sectional area of the tongue at both hard palate (from 1662 ± 311 to 1339 ± 254 mm^2^; *p* < 0.01) and pterygoid hamulae (from 1425 ± 222 to 1041 ± 150 mm^2^; *p* < 0.01), and 2 to 3-fold increase in cross-sectional area of the oropharynx at both hard palate (from 226 ± 68 to 595 ± 138 mm^2^; *p* < 0.01) and pterygoid hamulae (from 110 ± 64 to 351 ± 37 mm^2^; *p* < 0.01).

**Discussion:**

This study provides preliminary guidelines toward the feasibility and potential benefit of CMG in select cases of macroglossia-associated upper airway obstruction.

## Introduction

Artificial selection of genetic mutations linked to skull shape in dogs has resulted in breeds with varying degrees of facial retrusion, the shortened snout and widened hard palate common to brachycephalic dogs (1). These skeletal changes obstruct air flow in the nasal passages; however, the entire upper airway is further compromised by a relative excess of all the associated soft tissue structures which did not scale down proportionately with the skull, e.g., nostrils, turbinate mucosa, soft palate, tonsils and tongue (2).

Epidemiological studies have found that extreme brachycephalic breeds (Bulldog, French bulldog and Pug) die younger than other breeds, with a higher proportion of deaths related to upper respiratory tract dysfunction (3). Brachycephalic obstructive airway syndrome (BOAS) is a general term used to describe the full spectrum of structural and functional aberrations seen in some clinically affected brachycephalic dogs. Numerous primary anatomic abnormalities and deleterious secondary sequalae have been associated with BOAS (4, 5). Primary anatomic abnormalities include stenotic nares, elongated soft palate, aberrant nasopharyngeal turbinates, hypoplastic trachea, and macroglossia (5–11). Secondary sequelae include progressive laryngeal collapse, esophagitis, regurgitation, hiatal herniation, aspiration pneumonia, bronchial collapse, heart base tumors, altered blood parameters, and sleep apnea (6, 12–17). Consequently, numerous surgical procedures have been described to improve the airflow and welfare of these animals including alarplasty, staphylectomy, laryngeal sacculectomy, laser-assisted turbinectomy, and tonsillectomy (18, 19).

Interestingly, recent studies have determined that macroglossia may be a contributing factor to upper airway obstruction in dogs with BOAS (20, 21). Using computed tomography (CT), Jones et al. (20) demonstrated a relative macroglossia with reduced air volume in the upper airway in brachycephalic compared to mesaticephalic dogs, specifically at the level of the caudal hard palate and hamulae of the pterygoid bone. The conclusion of their study was that a relative macroglossia in brachycephalic breeds may contribute to upper airway obstruction. Seidenburg and Dupre used CT scans to compare total tongue volume and cross-sectional areas of the tongue, oropharynx, soft palate, and nasopharyngeal airways in three common brachycephalic breeds, pugs and French and English bulldogs. Their study noted that normalized tongue volumes and cross-sectional areas were smaller in the pug, and that this finding should be taken into account if surgical correction of a relative macroglossia is considered in this breed.

Very little information is available in the veterinary literature on the topic of tongue reduction glossectomy, e.g., feasibility and utility. Canine BOAS and human sleep apnea have many similarities, e.g., disordered breathing and episodes of oxygen desaturation associated with abnormal upper airway anatomy (9). Interestingly, caudal midline glossectomy (CMG) tongue reduction surgery is used for humans with severe sleep apnea persistent after failure of palatal surgery (22–24). Although CMG has a therapeutic role in human medicine, its potential role in relieving upper airway obstruction secondary to macroglossia in brachycephalic dogs remains undetermined.

To assess the feasibility and potential benefit in select cases of macroglossia-associated upper airway obstruction, the first objective of this study was to evaluate the effect of a standardized CMG procedure on tongue volume, as well as cross-sectional areas of the upper airway in brachycephalic dog cadavers. Additionally, we intended to assess the amount of caudal tongue muscle removed as well as proximity to the lingual artery.

## Materials and methods

Six donated, cadaveric brachycephalic dogs were used within 48 h of euthanasia. Dogs were held in a refrigerated room (4–8°C) prior to study and held at room temperature during setup time (approximately 2 h). None of the dogs had a clinical history of BOAS or prior airway surgery performed. All of the dogs were euthanized for conditions unrelated to BOAS. Upper airway and oral examinations were performed post-mortem to confirm the lack of prior airway surgery or obvious abnormalities of the airway or tongue, e.g., tumors.

**Figure 1:**
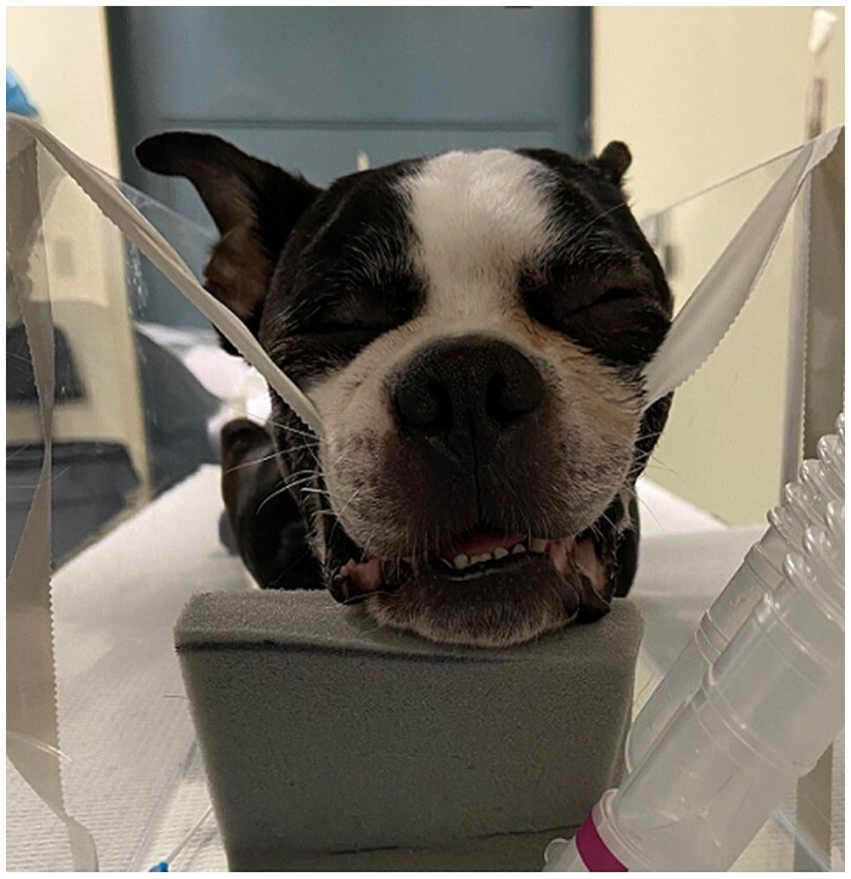
Positioning of dog for computed tomography. Note that the maxilla is suspended with tape behind the canines and the hard palate is parallel to the table. Radiolucent foam was used ventral to the rostral mandible to maintain consistent distance between upper and lower incisors.

Cadavers were positioned on the patient table using the same protocol: sternal recumbency, maxilla suspended with tape behind the maxillary canines and the hard palate parallel to the table. Initial positioning of the mandible and tongue for each dog was with the tongue retracted and the jaw nearly closed. Care was taken to maintain precise initial positioning throughout the experimental protocol for each cadaver, i.e., (1) between the rostral tongue and mandibular incisors, and (2) distance between the upper and lower incisors. Radiolucent foam was placed below the rostral mandible to maintain its position (Figure 1). Computed tomography was then performed of the entire skull. Following a caudal, midline reduction glossectomy, cadavers were replaced into the gantry in the exact same position and CT scans were repeated similarly.

### Computed tomography examination

A 16-slice CT scanner (Aquilion 16, Toshiba Medical Systems, Otawara, Japan) was used to perform all imaging. Volumetric data was acquired in 0.5-1 mm slice thickness from a level rostral to the nasal planum to a level caudal to the larynx by using a pitch of 0.69, 120 kVp, 100 mA, and a tube rotation time of 0.5 s. Sagittal and transverse images were reconstructed in either 0.5- (*n* = 1), 1- (*n* = 1), 2- (*n* = 3) or 3-mm (*n* = 1) slice thickness using a soft tissue algorithm.

Digital Images and Communication in Medicine (DICOM) files from the computed tomography were uploaded and segmented using Mimics Innovation Suite 26 (Materialise NV, Leuven Belgium) with thresholds set at −1,023 to −75 Hounsfield units (HU) and − 200 to 150 HU focused on soft tissue segmentation (20). Additional segmentation of the transitional areas was performed as previously described (20). Assessments of the total tongue volume and cross-sectional areas of the upper airway were performed as previously described (20, 21). Briefly, the tongue was segmented in all planes to ensure accurate volume rendering. Total tongue volume was measured according to the following guidelines for determining borders: ventrally-geniohyoid, mylohyoid, and hyoid muscles; dorsally-oral cavity and oropharynx; laterally-mandibular teeth, digastricus muscle, and hyoid apparatus; and caudally-basihyoid bone. Positioning was verified in transverse, dorsal, and sagittal planes. If the hard palate was not parallel to the CT table, reformatted transverse images were created within the Mimics software so that the transverse planes were perpendicular to the plane of the hard palate to ensure accurate cross-sections. Tongue, oropharynx, nasopharynx, and palatal soft tissue areas were evaluated at two transverse areas, the caudal hard palate (HP) and pterygoid hamulae (PH). HP was determined as the very last transverse CT slice in which the caudal nasal spine was visible. PH was determined as the very last transverse CT slice in which the hamulae of the pterygoid were visible. CT images were also used to measure the skull index (skull width/skull length ratio) to categorize the cadaveric skull conformation in our cadaveric dogs (25). The width was determined as the greatest distance between the outer borders of the zygomatic arches on transverse view. The length was determined as the most rostral aspect of the incisive bone by the caudal border of the occipital bone (dorsal border of the foramen magnum) on sagittal view.

### Caudal midline reduction glossectomy

**Figure 2:**
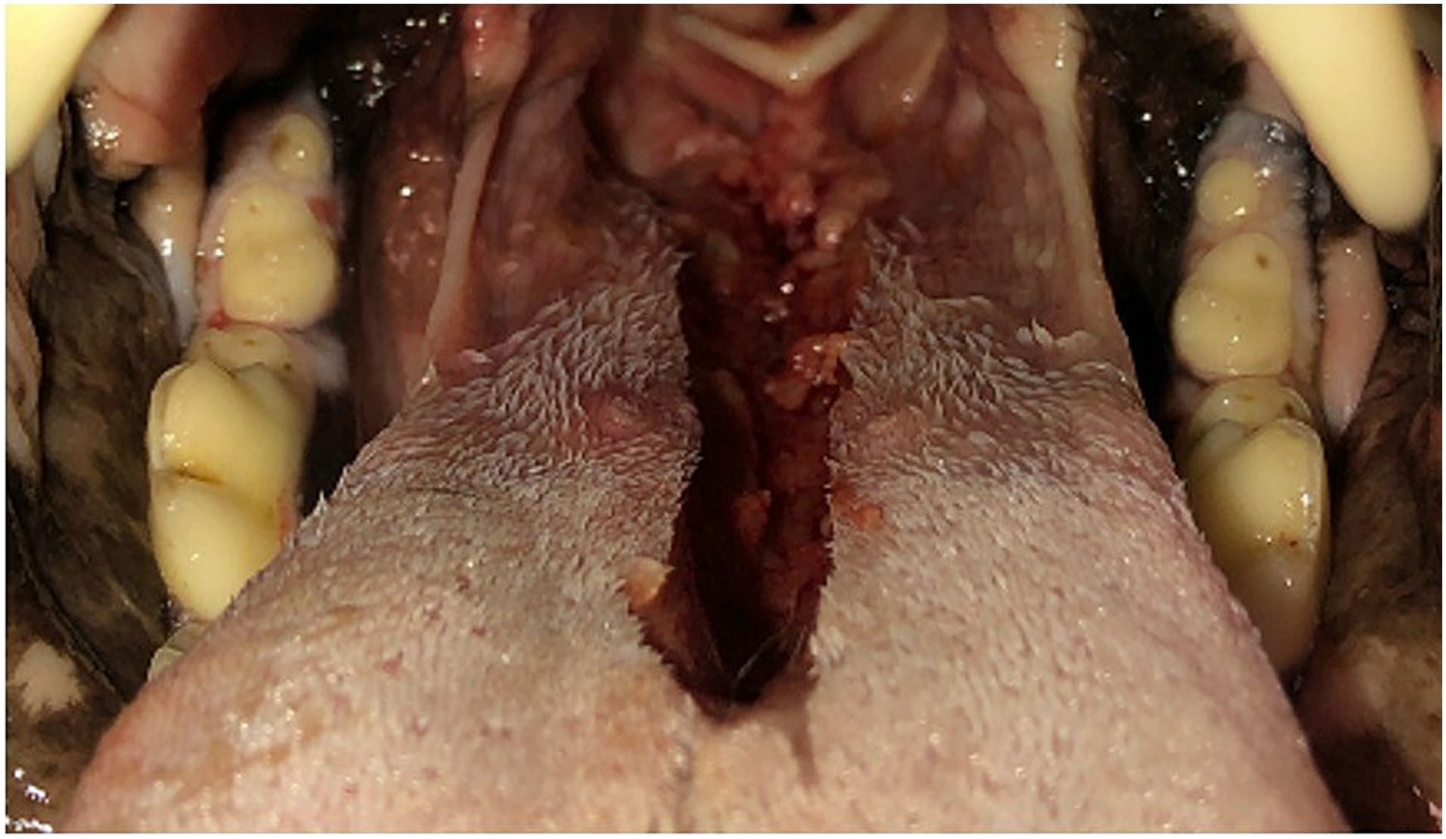
Caudal midline reduction glossectomy was performed beginning at the base of the epiglottis and extending rostrally approximately 60% of the tongue length.

The patient was placed in sternal recumbency with the maxilla suspended with gauze for visualization of the oral cavity. Caudal midline reduction glossectomy was performed (22, 23). A rectangular midline segment of tongue was excised using a #10 blade, beginning at the base of the epiglottis and extending rostrally approximately 60% of the tongue length (Figure 2). The CMG tissue dimensions used in this study were standardized (33% width and 50% depth of the caudal tongue and 60% of the total tongue length) and based on literature descriptions, initial CT measurements (sagittal and transverse images) and lingual artery anatomic position. The glottal defect was closed using a simple continuous suture pattern of 2–0 nylon suture. After completion of post-glossectomy CT imaging, the tongue was dissected from the cadaver and tissue measurements and weights were performed.

### Statistical analysis

Age, bodyweight, body condition score (BCS; 1–9 scale) and skull conformation data were reported with median values. Continuous numerical variables assessed included tongue volume and dimensions, and cross-sectional areas (nasopharynx, palatal soft tissue, oropharynx, tongue). Continuous variables were reported as mean ± standard error of the mean. Paired data were examined statistically for all comparisons. Comparisons between the pre-and post-glossectomy data were made by Student’s *t*-test, with *p* < 0.05 taken as the level for significance.

## Results

Brachycephalic breeds included English bulldog (*n* = 2), American bulldog (*n* = 1), Pug (*n* = 2), and Boston terrier (*n* = 1). The median age was 9.5 years, bodyweight was 13.4 kg, and BCS was 6.0. The median L: W index and skull index values were 1.09 and 0.92, respectively (Table 1).

**Table 1:** Signalment, bodyweight, body condition score, and skull conformation.

Breed	Gender	Age (years)	Weight (kg)	BCS (1–9)	Skull Length (cm)	Skull Width (cm)	L: W index	Skull index
English bulldog	MN	8.0	18.0	5.0	14.8	12.7	1.17	0.86
English bulldog	MN	9.0	26.0	7.0	13.3	14.3	0.93	1.08
American bulldog	F	9.0	29.8	6.0	16.4	13.1	1.25	0.80
Pug	F	15.0	7.0	6.0	9.1	8.9	1.02	0.98
Pug	M	14.0	8.3	6.0	9.5	8.6	1.10	0.91
Boston Terrier	M	10.0	8.7	5.0	10.8	10.0	1.08	0.93
Median		9.5	13.4	6.0	12.1	11.4	1.09	0.92

**Figure 3:**
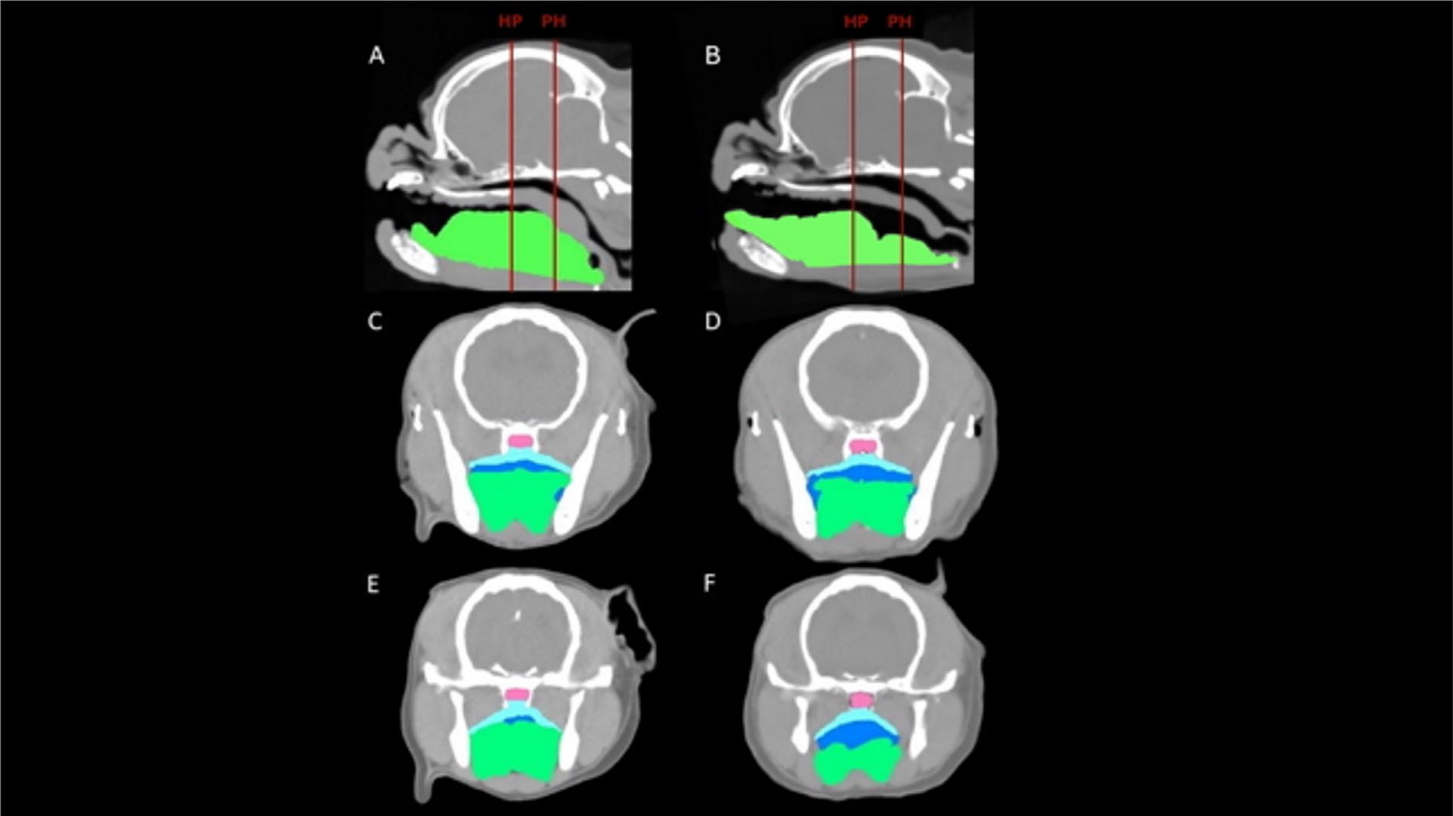
CT images of a representative cadaveric dog pre-glossectomy (left column) and post-glossectomy (right column). Midline sagittal images **(A,B)** illustrate the transverse levels at the caudal hard palate (HP) and pterygoid hamulae (PH) with red lines. Transverse images **(C,D)** are at the HP; Transverse images **(E,F)** are at the level PH. Highlighted areas represent the nasopharynx (pink), palatal tissues (aqua), oropharynx (royal blue), and tongue (green). In all images, note the decreased tongue tissue and markedly increased oropharyngeal air post-glossectomy (right) compared with pre-glossectomy (left).

The CMG resulted in a decrease (*p* < 0.001) in mean cross-sectional area of the tongue at both HP (20 ± 3%) and PH (25 ± 5%). Conversely, CMG resulted in a 2.5-fold increase (*p* < 0.005) in mean cross-sectional area of the oropharynx at HP and a 3-fold increase (p < 0.005) at PH (Figure 3). There was no significant difference in mean areas of the nasopharynx, palatal soft tissue or total area between pre-glossectomy and post-glossectomy at either level (Table 2).

**Table 2:** Cross sectional areas at hard palate and pterygoid hamulae.

Area	Hard palate			Pterygoid hamulae		
	Pre-glossectomy	Post-glossectomy	*p*-value	Pre-glossectomy	Post-glossectomy	*p*-value
Tongue (mm^2^)	1,662 ± 311	1,339 ± 254*	0.005	1,425 ± 222	1,041 ± 150*	0.009
Oropharynx (mm^2^)	225 ± 68	595 ± 138*	0.004	110 ± 64	351 ± 37*	0.001
Soft Palate (mm^2^)	145 ± 32	140 ± 36	0.751	358 ± 73	370 ± 73	0.224
Nasopharynx (mm^2^)	111 ± 25	106 ± 25	0.225	90 ± 19	98 ± 19	0.141
Total area (mm^2^)	2,143 ± 419	2,179 ± 426	0.178	1984 ± 344	1860 ± 263	0.253
Airway-total (mm^2^)	337 ± 93	701 ± 163*	0.005	201 ± 79	449 ± 53*	0.001
Tissue-total (mm^2^)	1806 ± 332	1,478 ± 273*	0.004	1783 ± 290	1,411 ± 219*	0.010
Airway/Tongue (%)	0.19 ± 0.04	0.53 ± 0.07*	0.001	0.13 ± 0.04	0.44 ± 0.03*	0.001
Airway/Tissue (%)	0.17 ± 0.03	0.46 ± 0.05*	0.0003	0.10 ± 0.03	0.33 ± 0.002*	0.002

Values are mean ± SEM.

*Significant difference from pre-glossectomy using paired Student’s *t*-test.

There was a 21 ± 3% decrease (*p* < 0.01) in mean tongue volume between pre-and post-glossectomy (Table 3). Mean tongue volume pre-and post-glossectomy for the bulldogs (*n* = 3) was 132,176 ± 18,543 and 106,920 ± 16,564 mm^3^, respectively. Mean tongue volume pre-and post-glossectomy for the pugs and Boston terrier (*n* = 3) was and 42,916.1 ± 3,736 and 33,599 ± 5,392 mm^3^, respectively. Mean tongue volume/bodyweight pre- and post-glossectomy for the bulldogs was 5,420 ± 243 and 4,367 ± 94 mm^3^/kg, respectively. Mean tongue volume/bodyweight pre- and post-glossectomy for the pugs and Boston terrier was 5,450 ± 884 and 4,277 ± 940 mm^3^/kg, respectively.

**Table 3:** Tongue volumes pre- and post-glossectomy.

Parameter	Pre-glossectomy	Post-glossectomy	*p*-value
Total tongue volume (mm^3^)	87,546 ± 21,121	70,259 ± 17,586*	<0.01
Tongue volume/bodyweight	5,435 ± 335	4,322 ± 346*	<0.001
Tongue volume/LW index	89,014 ± 20,647	71,011 ± 17,059*	<0.001
Tongue volume/skull length	724 ± 115	575 ± 99*	<0.002

Values are Mean ± SEM.

*Significant difference from pre-glossectomy using paired Student’s *t*-test.

There was no significant difference in inter-arterial (lingual) distance/tongue width pre- versus post-glossectomy at either HP (45 ± 2 versus 49 ± 4%) or PH (50 ± 2 versus 45 ± 4%). There was no significant difference in lingual artery depth/tongue depth pre- versus post-glossectomy at either HP (68 ± 2 versus 69 ± 1%) or PH (66 ± 3 versus 67 ± 5%) (Table 4).

**Table 4:** Lingual artery and tongue measurements from CT cross-sectional images.

Measurement	Hard palate				Pterygoid hamulae			
	Pre-glossectomy	Post-glossectomy	% reduction	*p*-value	Pre-glossectomy	Post-glossectomy	% reduction	*p*-value
Inter-arterial distance (mm)	22.9 ± 2.6	22.9 ± 2.4	−1.9 ± 7.9	0.982	24.9 ± 2.3	20.7 ± 1.8*	16.2 ± 3.0	0.004
Tongue width (mm)	50.3 ± 4.1	47.3 ± 3.9*	5.9 ± 1.6	0.018	50.2 ± 4.4	44.6 ± 4.2*	11.5 ± 1.3	<0.001
Arterial distance/Tongue width	45.2 ± 2.4	48.8 ± 3.9	−8.1 ± 7.4	0.280	49.6 ± 1.9	46.7 ± 1.0	5.4 ± 3.3	0.130
Lingual artery depth (mm)	21.1 ± 2.9	18.4 ± 2.4*	12.3 ± 3.9	0.032	18.9 ± 1.6	15.1 ± 0.8	18.1 ± 7.4	0.079
Tongue depth (mm)	31.0 ± 3.4	26.8 ± 3.6*	14.3 ± 3.8	0.003	28.7 ± 2.3	23.1 ± 1.9*	19.5 ± 0.7	<0.001
Arterial depth/Tongue depth	67.5 ± 2.2	68.9 ± 0.6	−2.5 ± 3.1	0.499	66.0 ± 3.1	66.5 ± 4.7	−2.0 ± 9.5	0.943

Values are mean ± SEM.

*Significant difference from pre-glossectomy using paired Student’s *t*-test.

Percent CMG tissue/total tongue data from excised tissue following study completion for weight, width, depth and length were 19 ± 2%, 35 ± 1%, 49 ± 1% and 53 ± 4%, respectively (Table 5).

**Table 5:** Excised tongue measurements and weights.

Measurement	Weight (grams)	Width (mm)	Depth (mm)	Length (mm)
Tissue	13.2 ± 3.4	17.0 ± 1.5	13.3 ± 1.5	67.0 ± 5.8
Tongue	71.2 ± 17.9	48.3 ± 3.8	27.0 ± 3.0	130.8 ± 14.3
Tissue/Tongue %	19.1 ± 2.1	35.1 ± 0.8	49.4 ± 1.4	52.7 ± 4.4

Values are mean ± SEM.

## Discussion

The current cadaveric study demonstrated a potential benefit for CMG to reduce tongue volume in dogs with macroglossia-related obstruction of the caudal oral cavity and pharynx. The procedure used in the study involved removing approximately 33% width and 50% depth of the caudal tongue (tongue root extending rostrally into body) and 60% of the total tongue length. This standardized glossectomy procedure averaged a 20% reduction in total tongue volume. From fixed, baseline positions of the tongue and mandible determined by experimental design, the midline glossectomy averaged a 20–25% reduction of the cross-sectional area of the caudal tongue within the pharynx. Additionally, the glossectomy width and depth kept the lingual artery at a reasonably safe distance.

The dimensions used for CMG in this cadaveric study are not provided as a universal recommendation for all clinical procedures to reduce the tongue, only as a solid frame of reference. Each case may have individual variations in tongue anatomy which will need to be accounted for using a pre-operative contrast CT for surgical planning. A CMG procedure is not described in the veterinary literature. Interestingly, descriptions of CMG techniques in human literature are often poorly detailed, i.e., do not explicate any parameters or guidelines for the extent of tissue excision. Limiting factors mentioned relate to the preservation of blood supply and muscular function of the tongue, i.e., lingual arteries and genioglossus muscle. The lingual arteries are lateral, medial and adjacent to the genioglossus, hyoglossus and inferior longitudinal muscles, respectively. Human literature describes the use of intra-op ultrasound or placing only superficial sutures to prevent damage to a lingual artery during CMG (22, 23). An additional surgical concern was the depth of the glossectomy be limited to prevent functional deficits of the genioglossus muscle and tongue movement (23, 24). The genioglossus is an extrinsic muscle which forms the bulk of the tongue (middle and ventral). Given the lack of specific guidelines in the literature, the extent of the standardized technique used in this cadaveric study was based on subjective assessment of diagrams and concern for genioglossus muscle function, the position of the lingual arteries on our transverse CT sections of the caudal tongue, and our CT sagittal views showing the prominent areas of dorsal incursion of the tongue into the pharynx and caudal oral cavity. It seems reasonable that a pre-operative contrast CT and similar guidelines would dictate the extent of CMG performed for a clinical case.

Mean L: W index and skull index for all the dogs in our study were within the range for brachycephalism, i.e., below 1.44 (26) and above 0.81 (27), respectively, but less severe than in recent clinical studies examining tongue volume and upper airway cross-sectional area measurements in brachycephalic dogs (20, 21). Skull index has been shown previously to have a significant association with BOAS status in pugs and bulldogs, but not all brachycephalic dogs, and other factors are involved (10). However, our data are consistent with such an association in that none of our dogs had a history of clinical signs, prior BOAS surgery or any stage of laryngeal collapse on post-mortem examination.

Cross-sectional areas of the tongue decreased (*p* < 0.01) and oropharynx increased (*p* < 0.01) following CMG at both levels of the pharynx. These changes resulted in an approximately 3-fold increase (*p* < 0.01) in airway/tongue and airway/tissue ratios at both the HP and PH. As discussed in Seidenberg and Dupre, no consensus exists regarding appropriate normalization methods for cross-sectional areas or tissue volumes of parts of the skull in small animals. Therefore, we included normalization indices used in prior studies (20, 21). However, an important consideration for interpreting or comparing upper airway cross-sectional areas is the beam angle of the CT relative to the rostro-caudal axis of the hard palate, tongue and mandible. Interestingly, these angles were markedly different between each of the recent brachycephalic airway studies, i.e., Jones et al. (20), Seidenberg and Dupre, and the current study. Briefly, Jones et al. (20) positioned the hard palate parallel to the table and angled the mandible and tongue downward. Seidenberg and Dupre held the mandible and tongue parallel to the table and angled the hard palate upward. In our study the hard palate was held parallel to the table, but the mandible and tongue were also placed near parallel. Each of these different head positions will result in different relative sizes of the adjacent areas of interest, i.e., the measured cross-sectional area will increase the farther the structure axis deviates from parallel to the patient table. Depending on what is being analyzed, standard or consensus positioning would be an important consideration for future studies.

Despite the positioning differences, it is interesting to compare the cross-sectional area proportion of air (oropharyngeal and nasopharyngeal)/soft tissue (tongue and palatal) at the hard palate and pterygoid hamuli levels between studies. The relative positions of the hard palate and mandible used in the current study, compared to tongue and airway measurements in prior clinical studies (20, 21), more closely resembled those of a dog with tongue retracted and jaw closed. Indeed, our incisor/mandibula opening angles pre- and post-glossectomy were 10.6 ± 1.1 and 10.7 ± 1.3 degrees, respectively, more acute than those reported by Siedenburg and Dupre (24.5 to 29.6 degrees). Thus, due to experimental design, our mean baseline air/soft tissue ratios at these levels (0.17 ± 0.03 and 0.10 ± 0.03) were much smaller than the averaged brachycephalic medians reported by either Jones et al. (20) (0.60 and 0.28) or Siedenburg and Dupre (0.54 and 0.26). However, after CMG our mean baseline air/soft tissue ratios at the HP and PH (0.46 ± 0.05 and 0.33 ± 0.01) were nearer to previously published data, especially those for the English bulldog (0.48 and 0.15) (21). Considering our baseline data, these latter comparisons are consistent with a potential beneficial effect of CMG to increase upper airway air/soft tissue ratios.

Our baseline tongue volumes were similar to prior studies, including breed differences (20, 21). After CMG, our group values for average tongue volume and volume/body weight ratio were 70,259 ± 17,586 mm^3^ and 4,322 ± 346 mm^3^/kg, respectively. It is worth noting that our mean volume/body weight ratio after CMG was similar to the median values previously reported for mesaticephalic breeds (4,454 mm^3^/kg) (20) and pugs (4,362 mm^3^/kg) (21).

Surprisingly, CMG resulted in no change in either lingual artery inter-arterial distance/tongue width or arterial depth from the dorsal surface of the tongue/tongue depth ratio measured at both the hard palate and pterygoid hamuli. In other words, any CMG related change in an arterial measurement occurred with a similar change in the corresponding tongue measurement. These corresponding changes are most easily recognized at the level of the pterygoid hamulus. For example, the decrease in pre- versus post-CMG inter-arterial distance (24.9 ± 2.3 versus 20.7 ± 1.8 mm) coincided with a decrease in tongue width (50.2 ± 4.4 versus 44.6 ± 4.2 mm). Importantly, the extent of the CMG used in the current study did not damage the lingual arteries in any of our cadavers, as assessed by the CT cross-sectional images and subsequent tongue dissection after each experiment. Although lingual artery position on CT cross-sectional images was determined from cadaveric tongues, their anatomic position is lateral and ventral to the genioglossus and intrinsic musculature, respectively, each readily identifiable on our transverse CT images. The methodology used to determine the position of the lingual arteries was reasonable, consistent and supported by tissue dissection. However, planning for and performing this procedure on a clinical patient would necessitate a thorough knowledge of tongue anatomy and a pre-operative contrast CT study including an accurate measurement of lingual artery position.

After completion of each study, the tongue was excised from the cadaver, tissue measurements were recorded, and the tongue was further dissected to check for lingual artery position and integrity. Notwithstanding dissection error and loss of anatomic position and moisture, examination of the excised tissues provided a reasonable assessment of relative proportions related to the CMG, i.e., removal of approximately 20% tongue volume, 33% width, 50% depth and 60% length (Table 5), and further dissection supported CT assessed lingual artery position and maintenance of integrity.

This cadaveric feasibility study has several limitations in addition to those already mentioned. It is not possible to directly extrapolate post-mortem measurements or results directly to performing the procedure on a clinical patient, as cadaveric tissues do not have identical properties or response compared to viable tissue. Additionally, we closed our cadaver CMG with a single layer, simple continuous suture pattern of 2–0 nylon instead of a multiple layer closure technique that would be used in a clinical procedure (28). Post-operative swelling, peri-operative management and clinical outcomes may preclude any apparent cadaveric utility or feasibility. However, this technique is used in humans and has been used successfully in a clinical case of macroglossia in a dog secondary to stable muscular dystrophy (RK). This study had a limited sample size (*n* = 6); however, results were paired, consistent and statistical significance was achieved. As with previous similar studies (20, 21), our imaging software required specific air and soft tissue areas to be identified on CT images of tissue. However, our studies were performed on cadavers within 24–48 h of euthanasia, and tongue structure delineation was consistently demonstrable. Prior to CT analysis, it was important to become familiar with the anatomy and relative positioning of tongue structures, e.g., with an imaging-based canine anatomy atlas and reviewing contrast CT images of clinical patients. We used consistent anatomical guidelines for each structure and area, similar to prior studies (20, 21). We also used similar methodology to minimize overlap and ensure that air was not included in soft tissue measurements and vice versa (20). Additionally, the methodology used in the current study was consistent for all dogs and, therefore, should not have markedly altered the data or deterred from the purpose of this feasibility study. Importantly, our CT findings were corroborated with tongue tissue dissection after the experimental protocol.

The findings of this study were consistent with CMG potentially being feasible and beneficial as an adjunctive surgical procedure in select BOAS patients, e.g., have Stage 1 laryngeal collapse, are minimally to non-responsive to standard surgical techniques, and have profound relative macroglossia. This technique could also be employed for other select clinical cases where macroglossia is causing moderate to severe dyspnea, or possibly dysphagia, e.g., a dog with stable muscular dystrophy. Although this procedure is used in humans and appears to be feasible and potentially beneficial for select dogs, its clinical use would require a pre-operative contrast CT and thorough knowledge of tongue anatomy. Additionally, peri-operative management must account for and accommodate temporary post-operative tissue swelling, i.e., compromised airway or ability to swallow. Given the apparent feasibility and potential benefits, the current study should help to establish reasonable guidelines and expectations when performing CMG. We believe that it was important to see the expected utility of this procedure in a cadaver prior to clinical use.

## Data Availability

The original contributions presented in the study are included in the article/supplementary material, further inquiries can be directed to the corresponding author.
